# Multi-scale dual-path attention network for seismic background noise attenuation

**DOI:** 10.1038/s41598-025-25446-x

**Published:** 2025-11-24

**Authors:** Li Han, Dongyan Wang, Feng Li

**Affiliations:** 1 College of Earth Sciences, Jilin University, Changchun City, Jilin China; 2 Department of Communication Engineering, Northeast Electric Power University, Jilin City, Jilin China

**Keywords:** Seismic exploration, Intense noise attenuation, Convolutional neural network (CNN), Multi-scale strategy, Attention mechanism, Weak signal recovery, Geophysics, Computer science

## Abstract

The background noise in seismic records severely interferes with the extraction of effective reflection events, particularly in complex exploration environments such as deserts. The non-Gaussian and nonlinear characteristics of background noise further exacerbate the difficulty of noise reduction, impacting the accuracy of subsequent processing such as inversion and migration. In recent years, deep learning has demonstrated excellent performance in the suppression of complex seismic noise, exhibiting notable advantages over traditional denoising algorithms. However, traditional deep learning networks often focus solely on feature extraction at a single scale, which proves inadequate when handling complex and variable seismic data. To address the aforementioned problem, we propose a Multi-scale Dual-path Attention Network (MSDPA-Net) aimed at enhancing denoising effectiveness by fully leveraging the multi-scale features of seismic data. MSDPA-Net employs a multi-scale strategy for preliminary feature extraction, followed by a dual-path attention module to discriminate between signal and noise. Subsequently, it utilizes a feature interaction structure for reinforcement learning, culminating in effective information fusion and reconstruction through a reconstruction module. Experiments on simulated and field seismic data demonstrate that MSDPA-Net exhibits remarkable performance in suppressing complex seismic noise compared to traditional denoising algorithms and typical deep learning networks.

**Supplementary Information:**

The online version contains supplementary material available at 10.1038/s41598-025-25446-x.

## Introduction

Seismic exploration stands as a significant technique in contemporary resource prospecting for underground reserves, where the precision and reliability of acquired data hold crucial importance for the effective development of resources. However, in practical exploration, the acquired seismic data unavoidably encounter various forms of noise interference, which not only obfuscate critical underground feature information but also severely impede subsequent data processing such as migration and interpretation^1^. It’s worth noting that various factors lead to the background noise in seismic data, with environmental noise at exploration sites being a primary source, including wind noise, surface vibrations and others^2^. Additionally, electronic components within exploration instruments can generate noise during operation. Furthermore, human activities such as transportation and industrial production also bring undesirable energy, thereby interfering with seismic data. Simultaneously, with the development of unconventional resource exploration, there is an upward trend in both the intensity and complexity of background noise in seismic data, presenting a serious challenge to the accuracy and utilization rate of seismic data^3^. Therefore, to accurately extract subsurface structural information and enhance the precision of exploration results, seismic noise attenuation is an indispensable step in seismic data processing.

To solve the above problems, data processors have proposed many classical denoising methods, which can be roughly categorized theoretically into five groups. The first one is the time-frequency filtering denoising methods^4^, which process data based on the differences between the effective signal and noise in the time and frequency domains. And then, noise suppression is realized by setting appropriate filtering parameters. Typical methods include bandpass filtering^5,6^, median filtering^7^, Wiener filtering^8^, and time-frequency peak filtering^9^. However, these methods show limited performance when confronted with aliasing noise. It means they are difficult to separate the signal and noise sharing the same frequency band^10^.

Sparse-transform-based methods utilize the difference in sparsity between signal and noise to achieve signal recognition, mainly including wavelet transform^11,12^, shearlet transform^13^, Curvelet transform^14,15^, seislet transform^16,17^. All these methods aim to accomplish the denoising task by telling the signal components from the noise in sparse domain. Nonetheless, the performance of these methods are directly impacts on the determination of threshold^18^.

The third category comprises low-rank methods, which regard the pure seismic data as a low-rank matrix, and the presence of noise increases the rank of the matrix; thus, suppressing seismic noise is equivalent to reducing the rank of the target matrix. Typical low-rank methods include Cadzow filtering^19^, singular spectrum analysis^20^, principal component analysis^21^, and robust principal component analysis^22^. Unfortunately, these methods may degrate when processed real seismic noise with non-Gaussian distributions^23^.

Decomposition-based methods decompose the noisy original seismic data into different modes. Noise removal can be achieved by analyzing all modes and selecting one or more modes that are most correlated with the effective signal for recombination, mainly including empirical mode decomposition^24^, variational mode decomposition^25^, and singular value decomposition^26^. Notably, the denoising accuracy may negatively influence by the aliasing modes. Taking EMD as an example, the aliasing modes contain both signal and noise components, while discarding or preserving them all adversely impact the denoising results^27^.

The last one is the denoising algorithms based on diffusion filtering, which can construct partial differential equations and adjust corresponding parameters to balance the effects of anti-diffusion and diffusion terms, so that it can effectively suppress the noise while maintaining the useful signals, among which the representative methods include the fractal conservation law (FCL)^28,29^ and so on. However, the determination of the threshold is always difficult, which impeding the further application of these methods^18,30^.

From the aforementioned descriptions, we can get the point that the tradictional methods all have their own limitations, although they have already been applied in field seismic data processing. Meanwhile, it is necessary to exploit and utilize the resources in complex geological structures with the depletion of easy accessible resources. Therefore, the quality of seismic data may significantly declined, and the performance and accuracy of these traditional methods may degenerate when coping with complicated and intense seismic noise.

In recent years, the continuous development of computer hardware has brought deep learning into focus. Deep learning, as a type of artificial neural network, can acquire deeper features and integrate them with shallow features to obtain distributed representations of data^31^, thereby reducing the workload of traditional feature extraction. The convolutional neural network represents a pivotal branch of deep learning, which has been extensively applied in seismic exploration, including microseismic detection^32,33^, geological structure detection^34,35^, seismic data inversion^36,37^ and noise suppression^38–40^. CNNs can identify the potential features in a directed way, enabling the completion of data processing tasks that traditional methods cannot achieve. With the continuous advancement of CNN theory, some classical CNN architectures such as DnCNN^41,42^ and Res-Net^43^ have been proposed and gradually applied to seismic data denoising. Compared to traditional methods, CNN-based approaches establish a nonlinear mapping between noisy and clean seismic records during network training, which can obtain effective signals with higher signal-to-noise ratios, more importantly, the method does not rely on strict assumptions and avoids manual parameter tuning. However, traditional CNN structures, such as DnCNN, have shallow network depths leading to insufficient extraction of weak signals. Res-Net effectively addresses the performance degradation in deep networks, however, due to its single structure, it cannot demonstrate excellent performance when dealing with low SNR seismic records.

The improvement and refinement of CNN have led to the gradual emergence of multiscale network structures. The strategy compensates for the limitations of conventional CNN frameworks by extracting and fusing multi-scale feature information so that the network can comprehensively utilize all the information and supplement the detailed components that may be lost. Seismic data contain multiple scales of information, including overall structures and local details. The large receptive field feature map can reflect the overall structure of seismic wave propagation paths and reflection interfaces in the media, which are crucial for seismic imaging and interpretation^44^. However, feature maps with large receptive fields generally have low resolution and cannot effectively capture detailed features. On the other hand, feature maps with small receptive fields can retain detailed information such as amplitude and phase, which are of great significance for seismic data denoising. There are primarily two methods to capture multiscale features. Firstly, by adjusting the data resolution through upsampling and downsampling, the input data can be transformed into samples of different sizes proportionally, followed by feature extraction at multiple scales using convolutional networks^45–47^. The second method uses convolutional kernels of different sizes to extract multiscale features while preserving the resolution of the image^48,49^.

To overcome the limitations of single-scale CNN, a Multiscale Dual-path Attention Network (MSDPA-Net) is designed in this paper to attenuate the noise in seismic data. MSDPA-Net utilizes a multi-scale strategy for preliminary feature extraction, followed by a dual-path attention module to discriminate between the signal and noise features. Subsequently, a feature interaction module facilitates reinforcement learning of the features. Finally, a feature reconstruction structure is employed to integrate and reconstruct effective information. MSDPA-Net can substantially improve the signal-to-noise ratio of seismic data by suppressing surface waves and random noise while preserving the effective signal. To assess the denoising performance of MSDPA-Net, it is compared with various competing methods, including traditional denoising algorithms and CNN-based methods. Results from synthetic and field data demonstrate that MSDPA-Net outperforms other comparative methods s in signal preservation and noise elimination.

## Network structure and denoising principle

Typically, the random noise in desert regions exhibits a relatively low dominant frequency. This phenomenon is attributed to the absorption effect of sand, which acts as the transmission medium for seismic waves. The effectiveness of traditional multi-scale-based denoising techniques have been verified in desert seismic data denoising. Yet, as deep learning continues to advance, CNNs are also being more widely used for this task, thanks to their outstanding denoising capabilities. To leverage the advantages of both approaches, we propose a new network that integrates multi-scale concepts with CNNs for seismic data denoising. Specifically, the backbone of our Multi-scale Dual-path Attention Network (MSDPA-Net) adopts a multi-scale structure to capture comprehensive multi-scale information. Next, a multi-scale feature extraction module and a feature interaction module are applied to integrate and enhance the captured multi-scale information. At the same time, a dual-path attention module is introduced to highlight key features, which further boosts the denoising performance. The detailed architecture of MSDPA-Net are described below.

### Architecture of MSDPA-Net

**Fig. 1:**
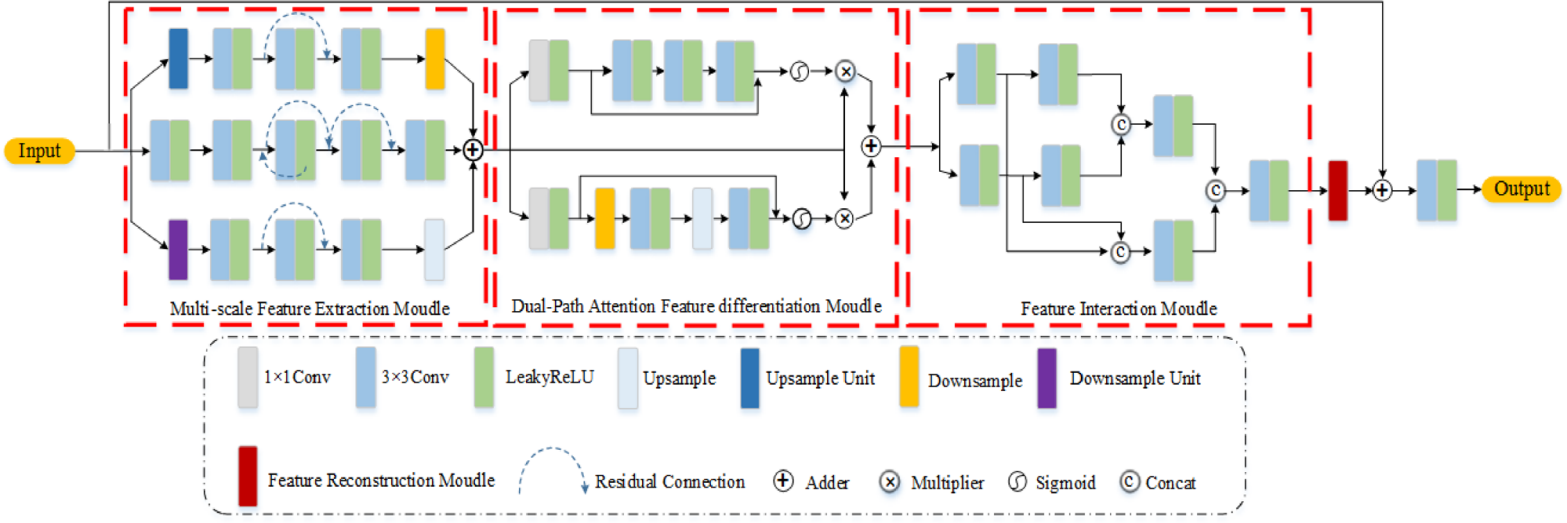
The overall architecture of the MSDPA-Net.

**Fig. 2:**
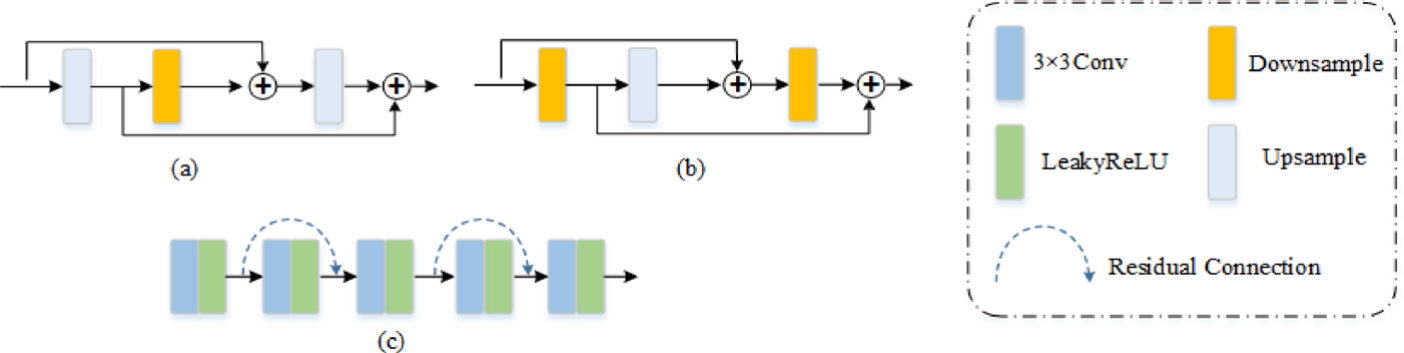
The basic structure of network components: **(a)** and **(b)** Upsample unit, downsample unit. **(c)** Feature reconstruction module.

To accomplish the task of denoising desert exploration data, we propose the MSDPA-Net denoising network, whose whole structure is depicted in Fig. 1. As we know, the patch size used for training and the size of the target data for application are always different; therefore, a fully convolutional neural network (FCNN) was employed instead of an ordinary CNN. We use a convolutional layer to preliminarily extract the potential features, and then the architecture of MSDPA-Net can be mainly divided into three modules. Firstly, MSDPA-Net employs a multi-scale strategy for initial feature extraction, in which both upsampling and downsampling units (Fig. 2(a) and (b)) are simultaneously introduced to efficiently extract and utilize original feature information. Meanwhile, the window size of the upsample/downsample layers is 2 × 2. Subsequently, the obtained information is fed into a dual-path attention module, which utilizes both scaled and unscaled pathways to respectively focus on enhancing and distinguishing features in the original and different scale feature maps. On this basis, a feature interaction structure is employed to facilitate interactive learning of the distinguished features from the previous process, enhancing the differentiation of characteristics between signal and noise. Finally, the feature reconstruction module is utilized to fuse and reorganize the signal features from these, resulting in the ultimate denoising outcome. Meanwhile, we use LeaKyReLU as the activation function, some continuous activation function, such as Swish or GeLU, may also be considered as potential candidates. The detailed descriptions for the main network components are explained as follows:

#### Multi-scale feature extraction module

In general, multi-scale feature extraction module has a parallel structure. At the high scale, convolutional kernels can capture local detail features, while at the low scale, they can extract global structural information. This multi-scale feature fusion helps to separate noise and effective signals more accurately. Specifically, the multi-scale feature extraction module introduces up- and down-sampling units, enabling the network to extract information features from three scales and effectively integrate feature information from different levels. The upsampling unit gradually restores the resolution of the feature map by alternating between a series of upsampling and downsampling operations, and uses an adder to extract and integrate low-resolution semantic information and high-resolution detail information. Similarly, during the downsampling process, the network gradually reduces the resolution of the feature map while increasing the number of channels of the feature map to extract low-resolution semantic information. This operation helps the network handle complex input data better and improve the generalization ability of the model. When the input data contains noise, the feature information at different levels can provide more context information for the network: High-resolution features may be more sensitive to noise, while low-resolution features can reconstruct the effective signals through the understanding of the overall structure. By fusing these features, the network can extract useful information more accurately and reduce the interference of noise on the output.

#### Dual path attention feature differentiation module

The designing purpose of dual-path attenuation feature differentiation module can be derived from the following three aspects: balancing between local details and global coarse information, improving the richness and accuracy of feature representation, enhancing the robustness and generalizability of the trained models. Specifically, it can distinguish between the signal and noise features initially obtained by the aforementioned multiscale structure, where the primary difference between the dual-path lies in whether there is a scale transform during the preceding feature extraction. The attention branch without scale transform relies on convolution to extract and fuse features, followed by a nonlinear transformation through the sigmoid activation function to further enhance the representation capability, paying more attention to local details and texture information in the input data. Due to the absence of scale transform, the network can maintain a higher spatial resolution, enabling more accurate localization of key areas. However, the absence of scale transform may also lead to difficulties in the network to capture the global contextual information. Meanwhile, the scale transform can be utilized to reduce the scale of the feature map, minimizing the amount of computation and storage space. By lowering the spatial resolution, downsampling can achieve the high-level representation of features while expanding the receptive field, thereby better characterizing long-range correlations between overall context and features. However, downsampling may lead to significant loss of important information, while upsampling cannot fully restore the missing data. MSDPA-Net integrates the above two, preserving both local details and overall structure, enhancing the richness and accuracy of feature expression. By combining the outputs of the two different pathways, MSDPA-Net can adaptively adjust the way features are extracted and fused based on different tasks and data characteristics, which improves the robustness and generalization of the model. Meanwhile, there is a complementary effect between the outputs of the dual paths, so that even if one pathway performs poorly in certain situations, the other pathway can still provide useful feature information, thus improving the overall stability of the model. Another notable matter is that it is appropriate to employ convolution-based partial attention because the network should handle data of various sizes (fully convolutional), although the size of the training data is small enough to apply full attention.

#### Feature interaction module

Generally, the feature interaction module aims to further improve feature extraction ability, thereby enhancing the denoising performance when confronted with intense aliasing seismic noise. In specific, it allows reinforcement learning of signal and noise features, making MSDPA-Net more efficient in distinguishing between effective signals and noise. The feature interaction module consists of a double-convolution block composed of two consecutive convolutional layers as the basic convolutional unit, which further explores hidden information from feature maps. Compared with traditional convolutional blocks, the double-convolution block has better feature extraction capabilities and a more compact network structure. As shown in Fig. 2(c), the feature reconstruction module consists of five convolutions with alternating residual connections, whose main function is to fuse and recombine the signal from the preceding network. Finally, skip connections are used to merge the features before and after the network, thereby accurately restoring weak effective signals.

### Denoising theory

In desert seismic exploration, effective signals are often obscured by various types of intense noise. Therefore, it is possible to linearly superimpose complex noise with pure signals to simulate the actual conditions of seismic data.

1 $$y=e+n$$

Where y represents the seismic record, e and n represent the effective signal of the substantial low-frequency noise present in desert records. MSDPA-Net constructs a nonlinear high-dimensional mapping F between the noise-containing exploration record and the effective signal, and the predicted effective signal can be expressed as:

2 $$\hat {e}=F(y,\eta )$$

Here $\eta =\{ w,b\}$ represents the network parameters, w is the weights, and b is the bias. To suppress the noise as much as possible while preserving the desired signal, the training process is optimized by choosing an appropriate loss function, denoted as follows:

3 $$l(\eta )=\frac{1}{{2B}}{\left\| {F({y_i},\eta ) - {e_i}} \right\|^2}$$

where B and ${\left\| \cdot \right\|^2}$denotes the batch size and L2 norm. In addition, the training data consists of pure signal blocks ${e_i}$ and noisy data blocks ${y_i}$. The optimal theoretical parameters can be obtained by decaying the loss function, denoted as ${\eta _{opt}}$. Subsequently, the final recovered signal ${\hat {e}_{opt}}$ is represented as follows:

4 $${\hat {e}_{opt}}=F(y,{\eta _{opt}})$$

## Construction of training set and setting of training parameters

### Generation of training set

To enhance the performance of the MSDPA-Net, it is equally crucial to ensure the quality of the training set. As network learning relies on high-quality training data, an incomplete or unrepresentative training set can lead to the learning of erroneous features^50^, thus affecting its denoising effect. Considering the confidentiality of seismic data, a training set comprising a combination of forward simulation data and real noise is established, providing a high-precision dataset that simultaneously encompasses both forward clean records and desert noise.

The construction of the signal set needs to fully consider the unique characteristics of seismic signals. The Ricker wavelet model is used to simulate four possible scenarios in actual seismic data, including oblique axis, hyperbolic axis, orthogonal axis, and fault axis, ensuring that the constructed dataset closely resembles the real seismic environment. Moreover, the locations of source varied across examples to enhace the diversity of generated records, therefore enhancing the variety of training data and improving the generalization of trained models. Table 1 shows the specific parameters of the forward modeling, and the definition of the Ricker wavelet is shown as follows:

5 $$f(t)=A\left\{ {1 - 2 \times {{\left[ {\pi {f_0}\left( {t - {t_0}} \right)} \right]}^2}} \right\} \times {e^{ - {{\left[ {\pi {f_0}\left( {t - {t_0}} \right)} \right]}^2}}}$$

Combined with the measured geological data, parameters in the forward modeling are adjusted to establish more and closer to real desert seismic records. On this basis, 100 different forward models are constructed to generate corresponding theoretical pure records by simulation. Subsequently, all simulated records are randomly cropped using a sliding window of size 64 × 64, resulting in a total of 17,408 valid signal patches. Finally, to ensure stability and convergence during the training process, these forward data are subjected to group normalization, forming a clean signal set suitable for denoising desert seismic data. As we know, the validation and testing procedures can enhance the generalization of the trained models. Generally, the signal and noise patches used to compose of validation and testing dataset are not included in training dataset. Furthermore, the vaildation dataset has similar properties, aiming to optimizing the setting of parameters; while we add some seismic patches with different features to constitute testing dataset, enhancing the generalization of the trained models. In summary, the training, validation and testing dataset are constructed with a ratio of 8:1:1.

**Table 1:** Physical parameters of the forward models.

Parameters	Specifications
Seismic wavelet	Ricker
Dominant frequency	15–37 Hz
Trace interval	20 m
Sampling frequency	2000 Hz
Wave velocity	700–5500 m/s
Number of source	1

**Fig. 3:**
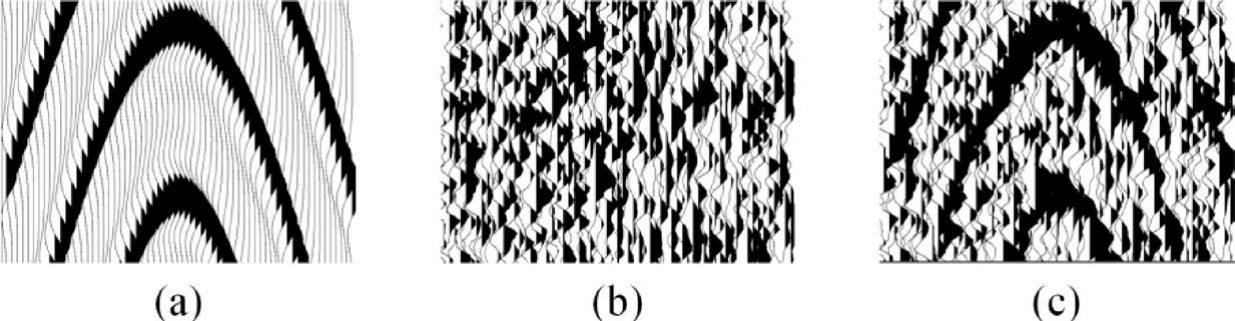
Generation of the training set. **(a)** A clean record patch. **(b)** A noise data patch. **(c)** A noisy record patch.

The field desert noise manifests as nonlinear, non-Gaussian, and non-stationary characteristics, which has a large impact on the effective signal. In this study, we use the noise data before first arrival to compose the noise dataset. Specifically, these seismic records only contain noise data without any reflection signals. Totally, we collect 2500 traces, and each trace is constituted of 1000 samples. Employing a sliding window of size 64 × 64, from which 25,000 noise slices are randomly intercepted and normalized to form a noise training set. Figure 3 illustrates an example of a training set.

### Setting of training parameters

The software platform utilized in this study is PyTorch. To meet the requirements of network training, all experiments were conducted on a computer equipped with a 3.20 GHz AMD Ryzen 7–5800 H CPU and NVIDIA GeForce RTX 3070 Laptop GPU. During network training, the batch size was set to 32, and the training patch size was 64 × 64. The L2 loss function was employed to calculate the loss value, and the Adam optimization algorithm was used to optimize the network. To facilitate gradient descent and prevent convergence to local minima, a learning rate decay strategy was designed, wherein the learning rate was reduced by a factor of 1/10 every 20 epochs. Additionally, the training samples were group normalized to ensure a uniform numerical range. The training period is set to 60 to ensure that the network has sufficient time to learn the features in the data. The specific training parameters are shown in Table 2.

**Table 2:** Parameters of MSDPA-Net’s network architecture.

Hyper-parameter	Specification
Optimizer	ADAM
Batch size	32
Patch size	64 × 64
Total layers	41
Learning rate range	[10^−4^,10^−5^,10^–6^]
Epoch number	60

## Processing of synthetic record

### Synthetic data processing

**Fig. 4:**
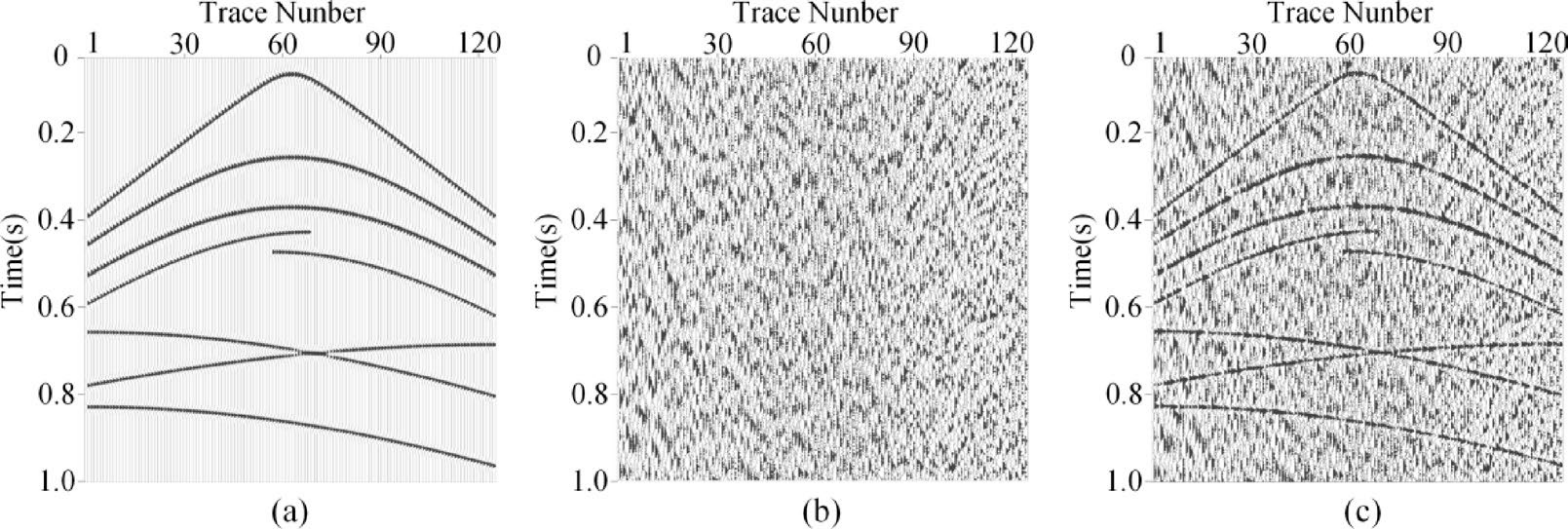
Generation of synthetic noisy record. **(a)** Clean record. **(b)** Field noise data. **(c)** Noisy synthetic record.

To verify the denoising capability of MSDPA-Net, synthetic seismic survey records are processed and analyzed in this section. For the construction of synthetic seismic exploration data, we employed a forward modeling approach to generate a pure synthetic record with a size of 1024 × 128, as shown in Fig. 4a, which consists of eight events with different waveforms and frequencies. By superimposing measured noise signals (Fig. 4(b)) onto synthetic signals, noisy data with an SNR of −5 dB were obtained as shown in Fig. 4(c).

**Fig. 5:**
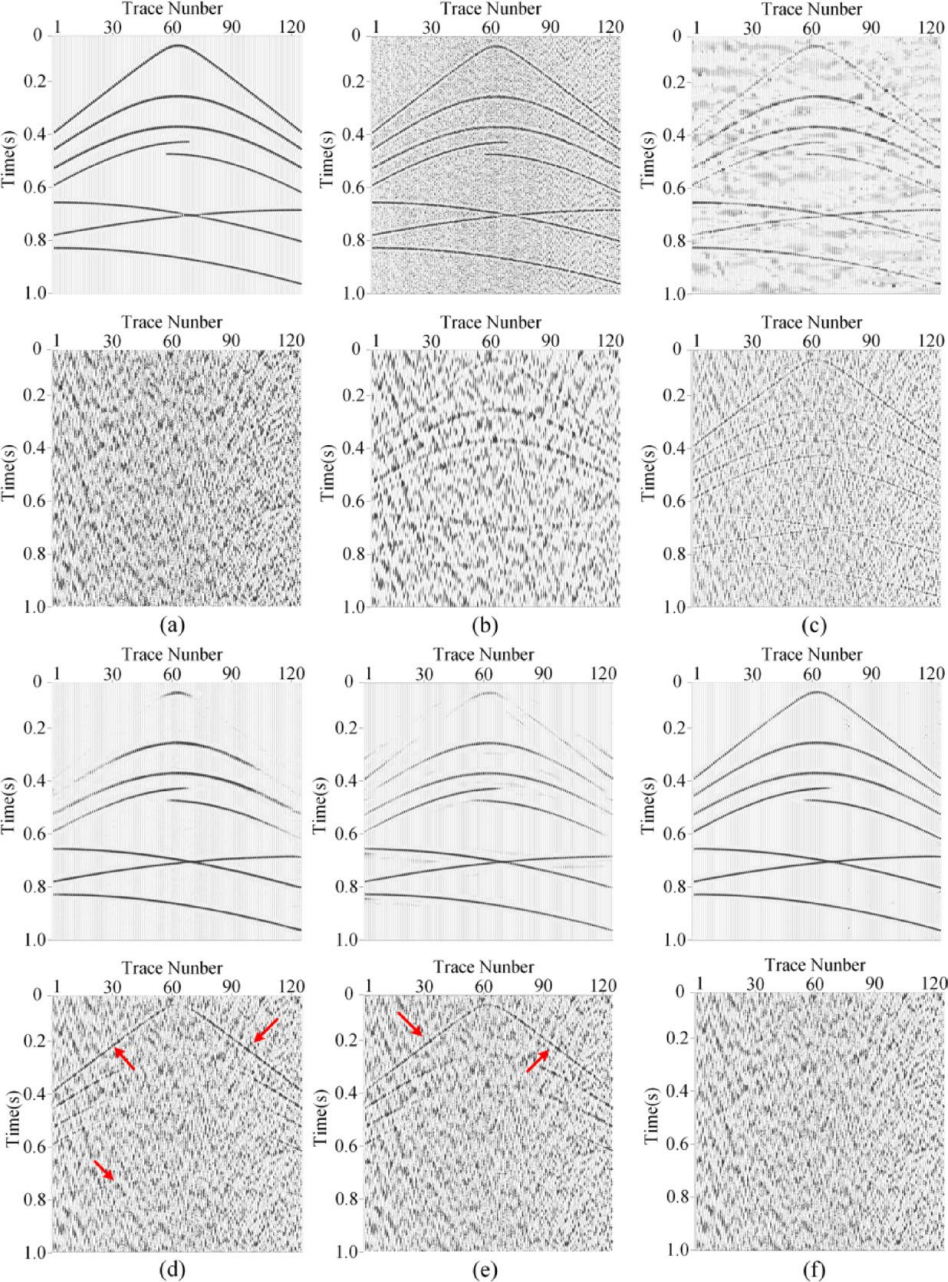
Performance of different denoising methods. **(a)** Clean synthetic data and the corresponding noisy record. **(b)-(f)** Attenuation results (top) and eliminated noise (bottom) garnered by TFPF, WT, DnCNN, U-Net, and MSDPA-Net.

In this paper, we evaluate the denoising performance of MSDPA-Net by comparing its denoising results with other methods, including two traditional methods, Wavelet Transform (WT) and Time-Frequency Peak Filtering (TFPF), as well as two deep learning methods, DnCNN and U-Net. The wavelet transform method uses db5 wavelet as the basis function with an 18-layer decomposition and employs soft thresholding for noise removal. In TFPF, the window length is set to 30. The contrastive methods based on CNN are trained in the same environment as MSDPA-Net, using the same dataset and similar hyperparameters. Table 3 lists the network parameters of DnCNN and U-Net, the constructed synthetic records are processed using the above methods, and the denoising results with filtered noise are shown in Fig. 5.

Observing the denoising results in Fig. 5 (a), it can be noticed that although TFPF retains the effective signal to a considerable extent, the presence of strong noise still affects the denoising results, leading to evident signal residues in the eliminated noise. On the other hand, as shown in Fig. 5 (b), WT almost fails to extract the effective signal from complex noise, and the recovered signal remains disturbed by noise interference. Compared to traditional methods, CNN-based methods exhibit better denoising capabilities. However, DnCNN and U-Net display unclear and discontinuous restoration of some events, with noticeable residual signals in the filtered noise, as indicated by the red arrows in Fig. 5 (d)-(e). In contrast, the results of MSDPA-Net are closest to the pure record, effectively suppressing low-frequency noise while restoring all events distinctly and coherently.

**Table 3:** Training configurations of DnCNN and U-Net.

Hyper-parameter	DnCNN	Res-Net
Optimizer	ADAM	ADAM
Batch size	32	32
Patch size	64 × 64	64 × 64
Epoch number	60	60
Learning rate range	[10^−4^,10^−5^,10^–6^]	[10^−4^,10^−5^,10^–6^]
Total layers	17	19
Convolution kernel size	3$\times$3	3$\times$3

### Frequency domain analysis

**Fig. 6:**
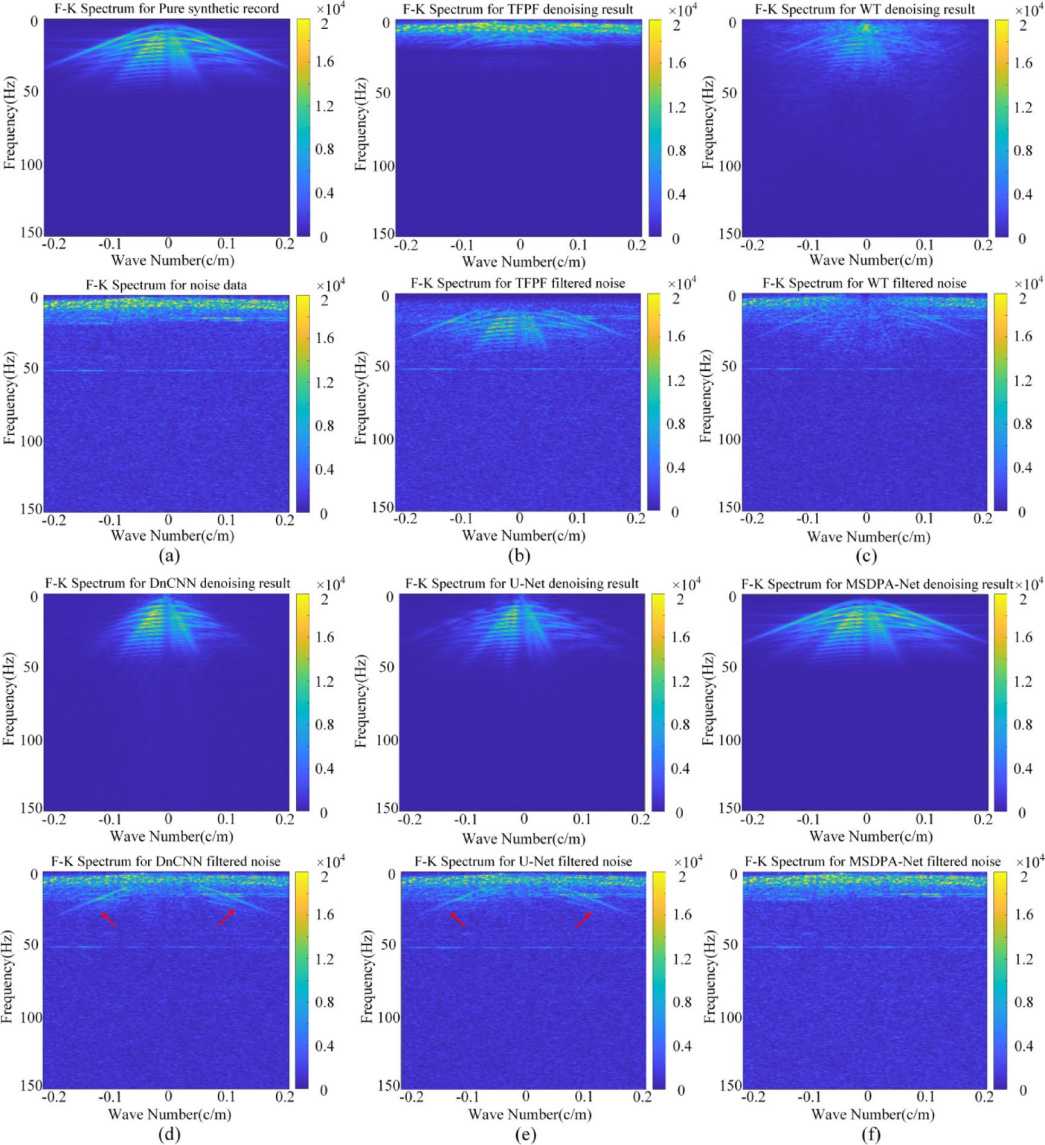
Comparisons of F-K spectral domain analysis. **(a)** F-K spectrum of the clean signal and the corresponding noisy data. **(b)-(f)** F-K spectra of the attenuation results (top) and the filtered noise (bottom) obtained by TFPF, WT, DnCNN,.

Spectrum analysis, as a crucial method for analyzing signal characteristics, provides a more intuitive representation of signal and noise properties compared to time-domain analysis. Therefore, to further evaluate the denoising effectiveness of MSDPA-Net, we further calculate and compare the F-K spectra of the results obtained by different methods in Fig. 5. As depicted in Fig. 6, from a frequency-domain perspective, the signal spectrum is primarily within the 5–50 Hz range, while the noise spectrum predominates in the 0–25 Hz range, with significant signal and noise aliasing in the low-frequency region, which has the same characteristics as the actual desert exploration data.

As shown in Fig. 6 (a), The F-K spectrum of the denoising result by TFPF (Fig. 6(b)) also contains a significant amount of noise components, while the recovery of the signal is also incomplete. There is a very obvious signal residue in the F-K spectrogram of the WT filtered noise in Fig. 6 (c), which indicates that the traditional methods are unable to obtain an effective signal from low-frequency noise. Similar to the conclusions drawn in the previous section, CNN-based methods exhibit superior performance in noise reduction. Both DnCNN (Fig. 6 (d)) and U-Net (Fig. 6 (e)) can separate desired signals from random noise. However, the discontinuity of their recovered signal spectra can still be observed in the F-K spectra of the denoising results. Moreover, a more obvious signal component can be observed in the F-K spectra of the filtered noise as shown by the red arrows in the figure, which indicates that DnCNN and U-Net still lose some signal energy during the denoising process. Overall, MSDPA-Net demonstrates the best denoising performance as shown in Fig. 6 (f), with the F-K spectrum of the recovered signal closely resembling that of the clean one. Additionally, there is no significant valid information present in the filtered noise, indicating that MSDPA-Net can maintain the integrity of the signal while eliminating background noise, achieving high-precision reconstruction of seismic events.

### Quantitative analysis

In this section, different methods are quantitatively evaluated by computing the SNR and root mean square error (RMSE). In general, the signal-to-noise ratio is a metric used to quantify the ratio of relative intensity or power between useful information and noise. RMSE, on the other hand, is commonly used to measure the deviation between the output and the label, where a smaller value indicates less loss of signal energy, reflecting better denoising performance. The definitions of SNR and RMSE are as follows:

5 $$SNR(dB)=10{\log _{10}}\left( {\frac{{\sum\nolimits_{{i=1}}^{N} {\sum\nolimits_{{j=1}}^{M} {u{{\left( {i,j} \right)}^2}} } }}{{\sum\nolimits_{{i=1}}^{N} {\sum\nolimits_{{j=1}}^{M} {{{\left[ {u\left( {i,j} \right) - v\left( {i,j} \right)} \right]}^2}} } }}} \right)$$

6 $$RMSE=\sqrt {\frac{1}{{MN}}\sum\nolimits_{{i=1}}^{N} {\sum\nolimits_{{j=1}}^{M} {{{\left[ {u\left( {i,j} \right) - v\left( {i,j} \right)} \right]}^2}} } }$$

We constructed multiple sets of noisy data and independently established a test noise set to avoid mutual interference between training and testing. TFPF, WT, DnCNN, U-Net, and MSDPA-Net were used to process the aforementioned data, and quantitative evaluations were performed using SNR and RMSE. As shown in Table 4, conventional methods failed to achieve the expected results, with limited improvement in SNR. Compared to other CNN methods, MSDPA-Net exhibited significant advantages in handling the radon noise, with both higher SNR improvement and greater RMSE reduction. Taking the − 8dB noisy record as an example, MSDPA-Net was able to increase the SNR by over 19dB, while the improvement effect of U-Net was only around 15 dB. The results of the quantitative analysis fully confirm the outstanding performance of MSDPA-Net in reducing desert seismic noise, effectively improving the quality of seismic data.

**Table 4:** Comparison of SNR and RMSE across various Attenuation algorithms.

Original record/dB	TFPF		WT		DnCNN		U-Net		MSDPA-Net	
	SNR	RMSE	SNR	RMSE	SNR	RMSE	SNR	RMSE	SNR	RMSE
0	3.60	0.0232	6.09	0.0131	9.11	0.0065	9.39	0.0061	14.74	0.0016
−2	2.02	0.0334	4.86	0.0173	8.72	0.0071	9.15	0.0065	14.46	0.0017
−4	0.30	0.0496	3.65	0.0229	8.15	0.0081	8.78	0.0070	13.79	0.0019
−6	−1.51	0.0752	2.44	0.0303	7.37	0.0097	8.24	0.0080	13.14	0.0021
−8	−3.39	0.1159	1.17	0.0406	6.31	0.0124	7.53	0.0094	11.75	0.0036

**Fig. 7:**
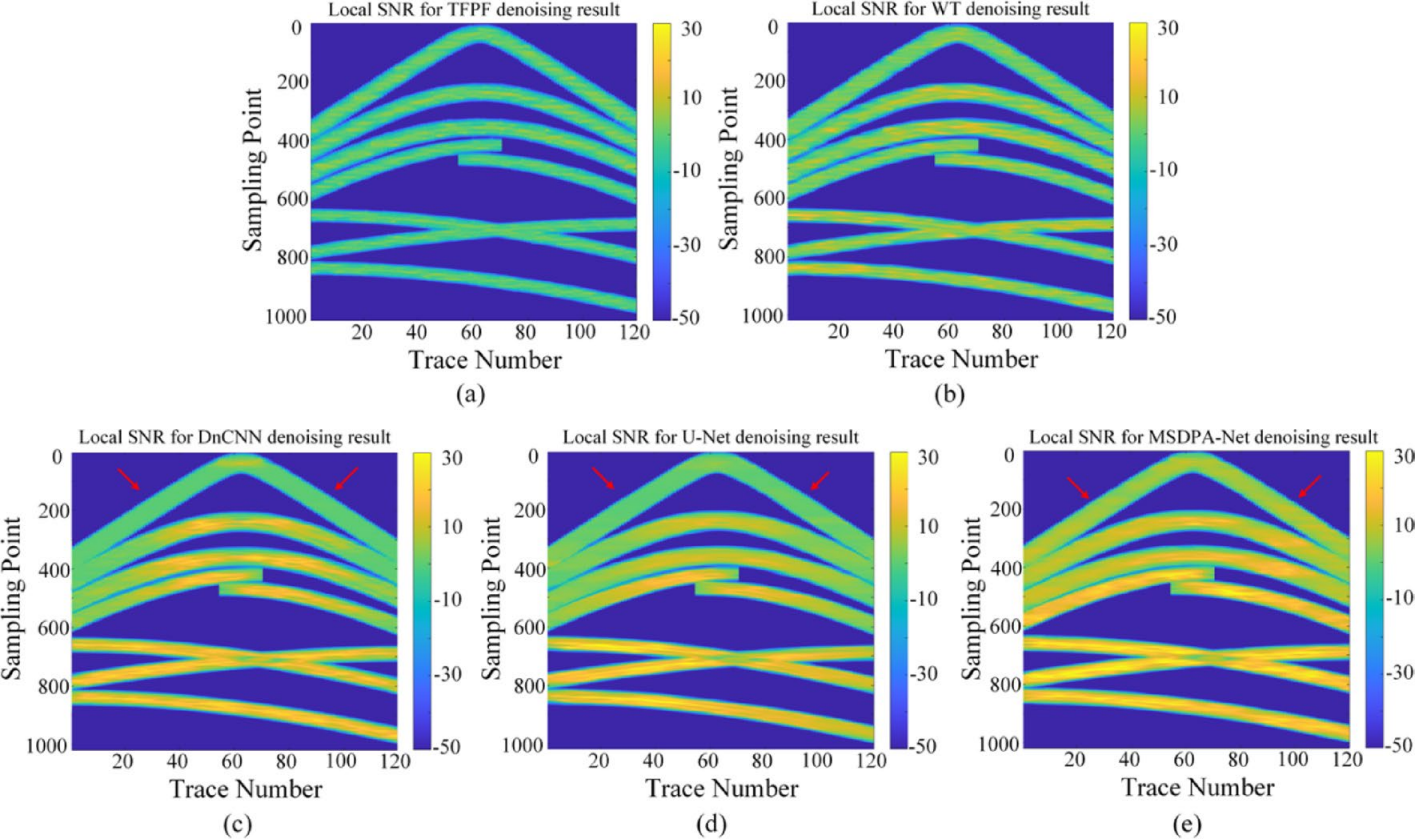
Local SNR comparison results. **(a)-(e)** Results of TFPF, WT, DnCNN, U-Net, and MSDPA-Net, respectively.

Meanwhile, to visually observe the SNR processed by each method, the local SNR of different denoising results was calculated. Specifically, using a sliding window with a stride of 5 to slice the denoising results, the local SNR of each slice was calculated according to the SNR formula. Generally, the brightness of colors in the image can intuitively reflect the energy intensity of the signal. The comparison of local SNR for all processing results is shown in Fig. 7. The energy of the events is lower in TFPF and WT (as shown in Fig. 7(a) and (b)), with almost no yellow areas in the results. In contrast, CNN-based methods can provide higher SNR, but from the results obtained from DnCNN and U-Net, it can be seen that some areas of the events have significantly lower SNR than MSDPA-Net, as indicated by the red arrow in Fig. 7(c)-(e). Overall, MSDPA-Net can provide a higher increment in SNR for desert seismic records, further validating its excellent performance in noise attenuation and signal preservation.

### Computational overhead analysis

For deep learning methods, computational efficiency is also an important factor to consider, so we analyze the training time and processing time of different methods. Typically, the computational complexity of the network is mainly influenced by the number of layers and channels. Since MSDPA-Net contains multi-scale and attention structures, it requires more convolutional layers and channels to extract information at different scales. According to calculations, the network parameters of MSDPA-Net, U-Net, and DnCNN are 1,320,801, 31,372,417, and 544,177, respectively, with training times of 5.4, 3.2, and 2.7 h. The 40 records generated are processed by using the above mentioned methods and evaluated for their performance, and the specific results are shown in Table 5. Although the training time is longer, MSDPA-Net is more effective and advantageous in terms of average SNR improvement. Despite the longer training time compared to traditional methods, deep learning algorithms reduce requirements for human intervention without the need for tedious fine-tuning processes and parameter settings. Furthermore, due to the excellent generalization ability of deep learning methods, the obtained models can be effectively applied to seismic records similar to the training dataset, thereby achieving high-quality denoising effects. Additionally, with the improvement of computing devices, the training cost of the network will also decrease, making the time cost required for applying MSDPA-Net acceptable.

**Table 5:** Comparison of computational cost and improved SNR for different methods.

Hyper-parameter	TFPF	WT	DnCNN	U-Net	MSDPA-Net
Processing time(s)	0.0113	0.0357	0.4992	0.5394	1.2220
Training time(hour)	-	-	2.7	3.3	5.4
Average improved SNR(dB)	4.20	7.64	11.93	12.62	17.87

### Effectiveness analysis of network components

**Fig. 8:**
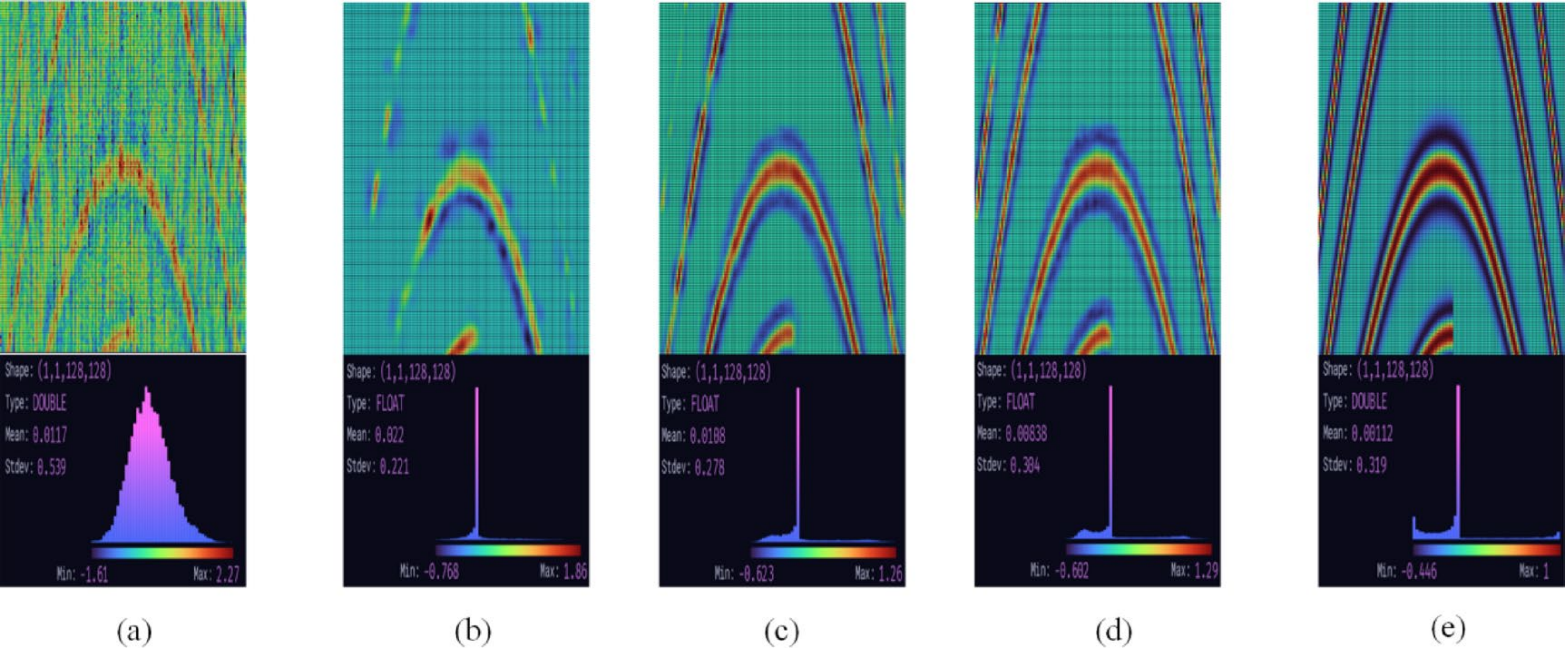
Visualization of feature maps at different stages. **(a)** Input. **(b)** to **(d)** Feature maps after multi-scale feature extraction module, attention module and feature interaction module. **(e)** Output.

In this section, we have extracted the feature maps for visualization analysis, which is shown in Fig. 8. We select the feature maps after multi-scale feature extraction module, attention module and feature interaction module to investigate the contributions of the network components, which are shown in Fig. 8(b) to (d), respectively. Notably, MSDPA-Net can effectively eliminate the intense background noise by comparing the input and output, shown in Fig. 8(a) and (b). Moreover, each network component is helpful for the denoising task, which has been verified by the gradually improving feature maps. It is shown that the proposed framework can effectively refine the informative features and attenuate the noise influence.

## Experimental analysis of field data

**Fig. 9:**
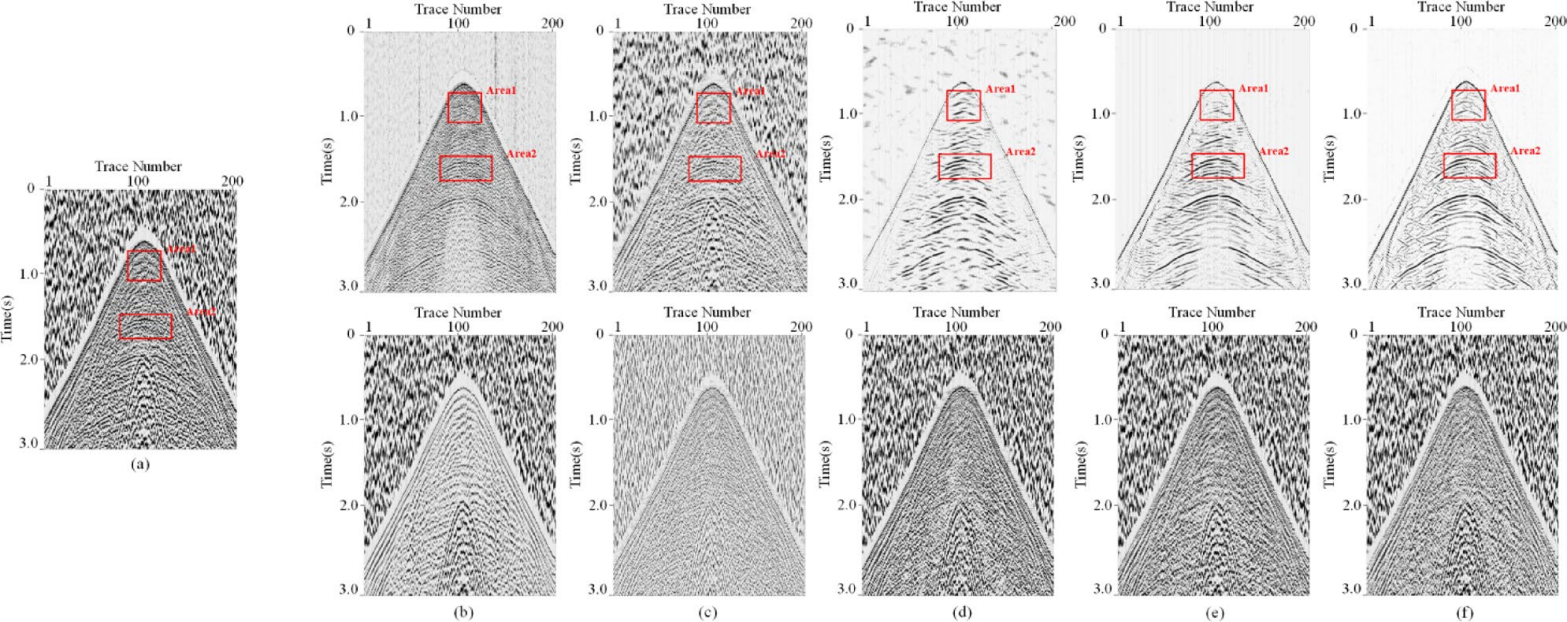
Comparative analysis for attenuation results of different algorithms for the field data. **(a)** Field seismic record. **(b)-(f)** Attenuation results of TFPF, WT, DnCNN, U-Net, and MSDPA-Net, respectively.

To verify the application performance of MSDPA-Net in field records, we selected a common-shot-point field desert seismic data for testing, which consists of 208 traces, with each seismic trace containing 1488 samples. As shown in Fig. 9 (a), this record exhibits strong noise energy, with significant amounts of random noise and surface waves obscuring the effective signal, resulting in some seismic events being truncated, severely affecting the continuity and identification of the effective signal. Using the five methods discussed in the previous section to process the field desert record, the obtained results are shown in Fig. 9 (b)-(f).

**Fig. 10:**
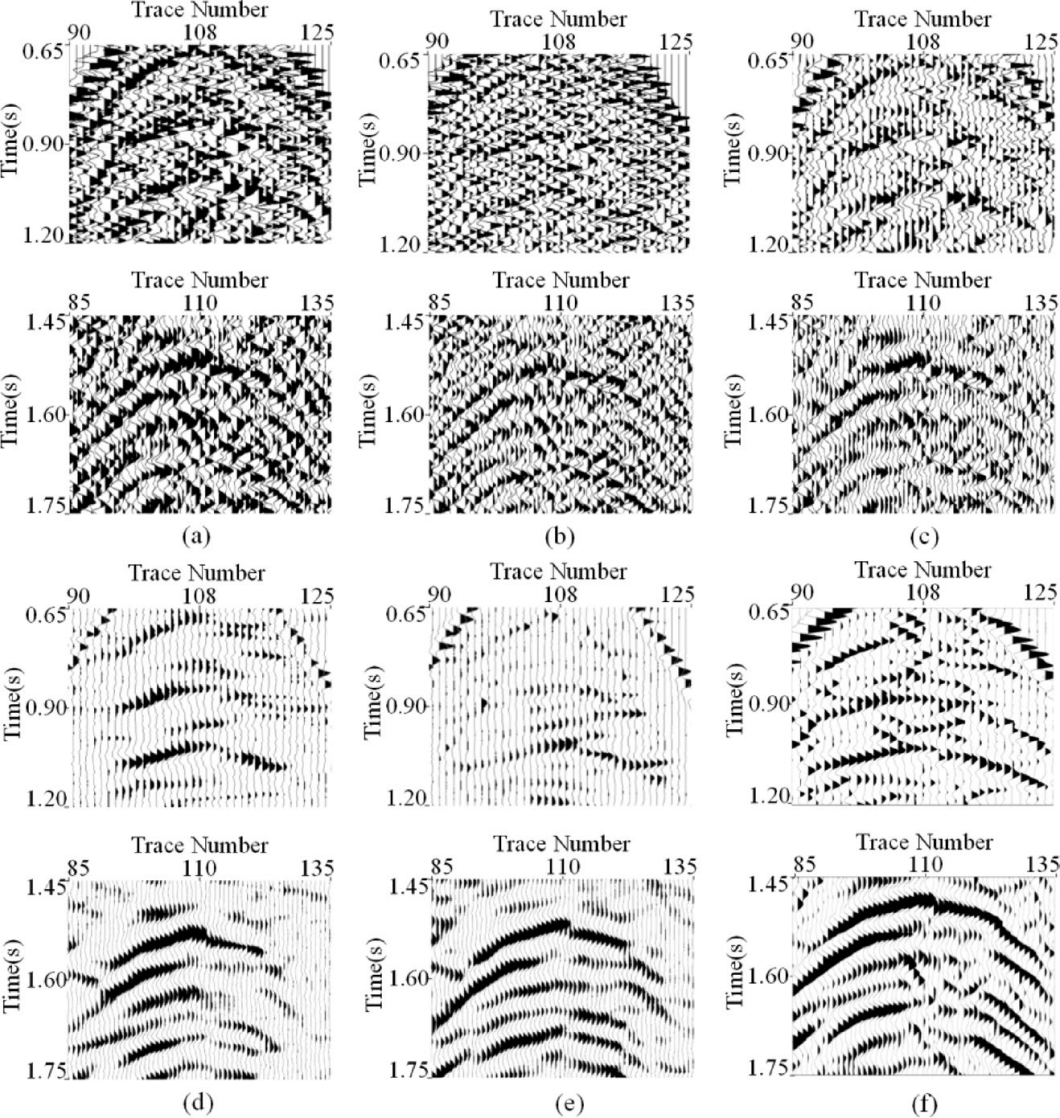
Enlarged comparison results for Area 1 (bottom) and Area 2 (top) in Fig. 8. **(a)** Field seismic record. **(b)-(f)** Attenuation results of TFPF, WT, DnCNN, U-Net, and MSDPA-Net, respectively.

As shown in Fig. 9 (b)-(c), Neither TFPF nor WT can effectively suppress the noise in the field records, the background noise in the denoised images still severely compromises the clarity and continuity of the effective signal. Although DnCNN has a certain effect in reducing intense interference in Fig. 9 (d), the result still contains some background noise, and there is no significant improvement in the continuity of the seismic events. As shown in Fig. 9 (e), U-Net can effectively remove most of the low-frequency noise but poorly preserves the amplitude of the effective signal. In contrast, MSDPA-Net not only effectively suppresses most of the random noise and surface waves but also maintains the integrity and continuity of the effective signal, providing support for subsequent geological analysis. To better observe the recovery capability of each method for seismic events, two regions highlighted by red boxes in Fig. 9 were magnified, and the local magnification results are shown in Fig. 10. TFPF and WT exhibit poor suppression of desert noise, and the effective signal remains difficult to identify. Although DnCNN and U-Net show significant improvements in denoising, they also exhibit some unclear recovery of seismic events, especially in the restoration of shallow information. In comparison, MSDPA-Net performs excellently in processing real seismic records, providing clearer and more continuous recovery of the effective signal.

**Fig. 11:**
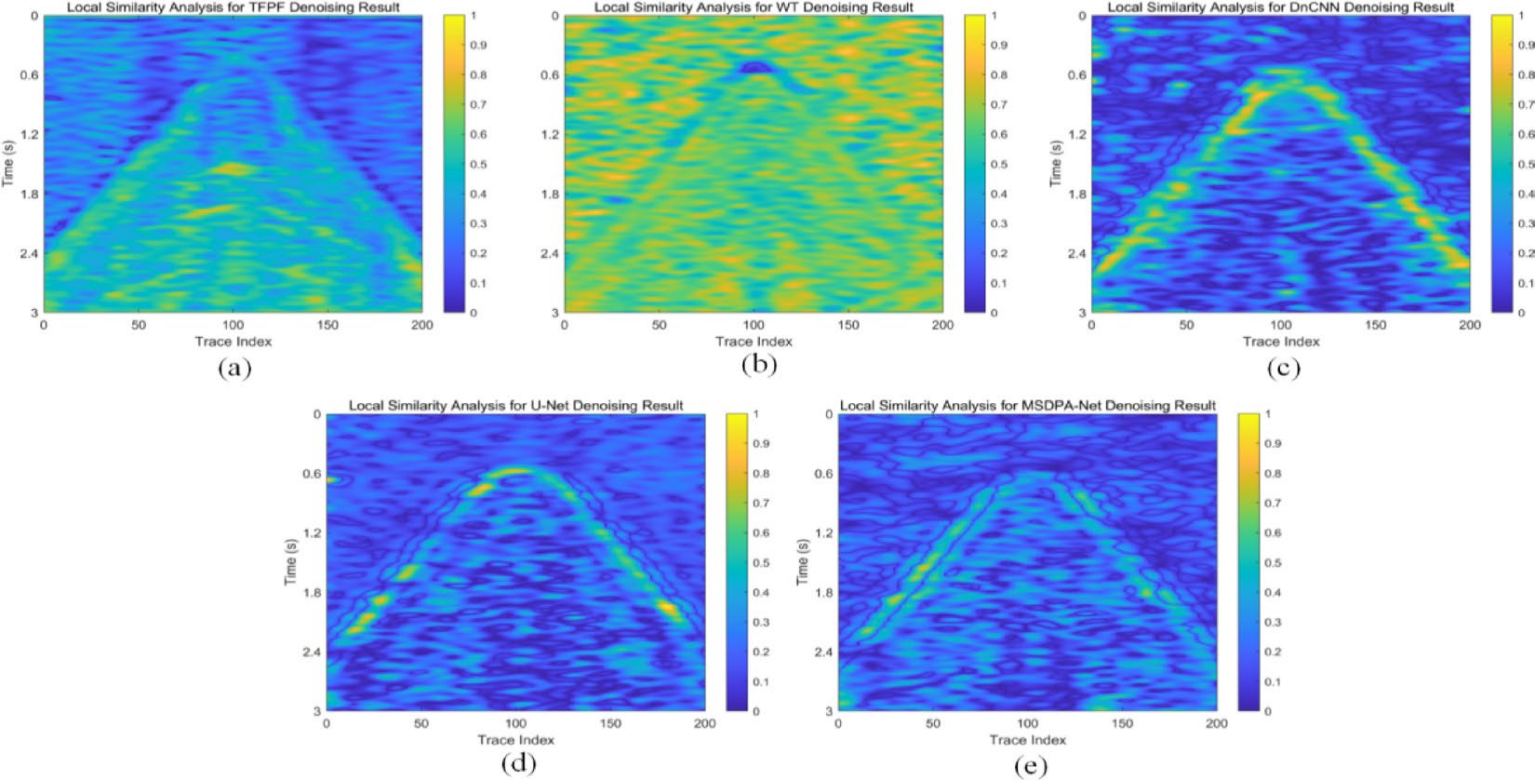
Local similarity between denoising results and filtered noise. **(a)** TFPF. **(b)** WT. **(c)** DnCNN. **(d)** U-Net. **(e)** MSDPA-Net.

Moreover, the signal leakage is also analyzed by investigating the similarity between denoising result and filtered noise. Here, we use the local-similarity proposed by Chen and Fomel^51^ to accomplish the task, and the corresponding results for different methods are shown in Fig. 11. Notably, obvious signal leakages are existed in the denoising results of TFPF and WT, as shown in Fig. 11(a) and (b). Meanwhile, the results of DnCNN and U-Net (Fig. 11(c) and (d)) depict signal leakage, reflecting by the large similarity value. Compared with other methods, MSDPA-Net (Fig. 11(e)) shows the limited signal leakage, proving its performance in preserving weak signals.

**Fig. 12:**
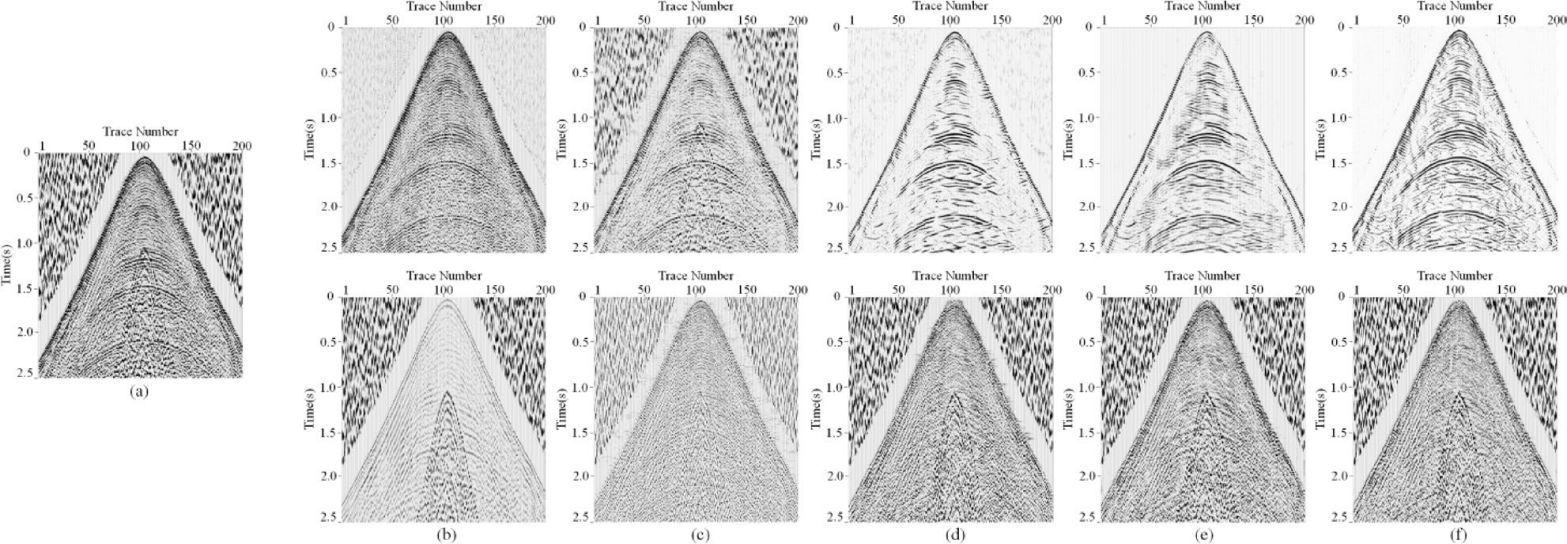
Processing result for another field seismic data. **(a)-(f)** Field data and the denoising results of Attenuation results of TFPF, WT, DnCNN, U-Net, and MSDPA-Net, respectively.

Meanwhile, to confirm the generalization performance of the proposed MSDPA-Net, another field seismic record was used for experimentation, as shown in Fig. 12 (a). The complexity of effective signals in this record is higher than that in Fig. 12 (a), posing higher requirements for denoising algorithms. Similarly, TFPF and WT show deficient suppression of random noise in Fig. 12 (b)-(c), and surface waves cause significant interference to the effective signal, making the events difficult to distinguish. As shown in Fig. 12 (d), Although DnCNN has improved in noise attenuation, there is still a considerable amount of low-frequency noise residue. The U-Net in Fig. 12 (e) can effectively reduce various types of noise but has significant deficiencies in maintaining the energy and continuity of the effective signal. As shown in Fig. 12 (f), the MSDPA-Net algorithm almost eliminates most random noise and surface waves, maintaining a high level of denoising performance while preserving the energy and continuity of the effective signal. In means that trained models of MSDPA-Net (without further retraining procedure) can apply to the seismic data acquired in the similar environments, demonstrating a certain generalization ability.

## Discussion

### Contributions of network components

**Fig. 13:**
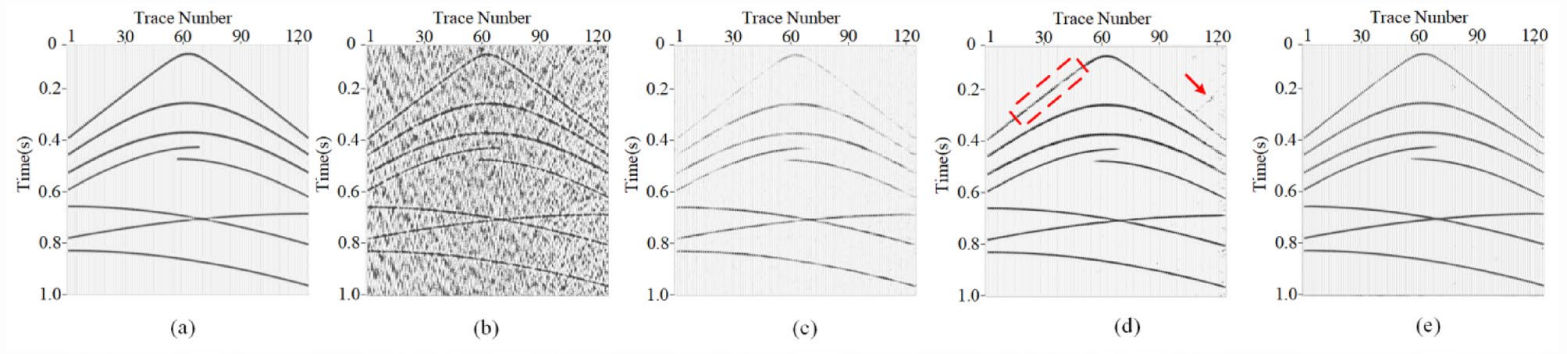
Processing results after different processing module in MSDPA-Net. **(a)** and **(b)** Clean and noisy synthetic record. **(c)** and **(d)** Denoising results after MSFE and DPAFD module. **(e)** Final results obtained by MSDPA-Net.

In this section, we aim to evaluate the contributions of each network component. In the designing of MSDPA-Net, we plan to capture the multi-scale features and improve the denoising capability. Therefore, the main framework of MSDPA-Net is composed by multi-scale feature extraction (MSFE) module, dual-path attention feature differentiation (DPAFD) module and feature interaction module. The main backbone of each module is determined, and we have changed the number of convolutional layers to balance the denoising performance and computational cost. Moreover, we have analyzed the output after each module to analyze the contribution and effectiveness of network components. The corresponding results are shown in Fig. 13. Figure 13(a) and (b) give the clean synthetic data and noisy record. We can find that the reflection events are seriously affected by the intense background noise. MSFE module, composed of 11 convolutional layers, can attenuate the background noise to some extent; however, the recovered events are blured. On the basis, DPAFD module is applied to further attenuate the intense noise, and the denoising result is shown in Fig. 13(d). Compared with the final denoising results (shown in Fig. 13(e)), that after DPAFD module still suffers from residual noise (marked by the red arrow) and discontinuous events (indicated by the red box). Therefore, we can get the point that the network components all contribute to the denoising performance of MSDPA-Net.

### Comparing with other state-of-art denoising methods

**Fig. 14:**
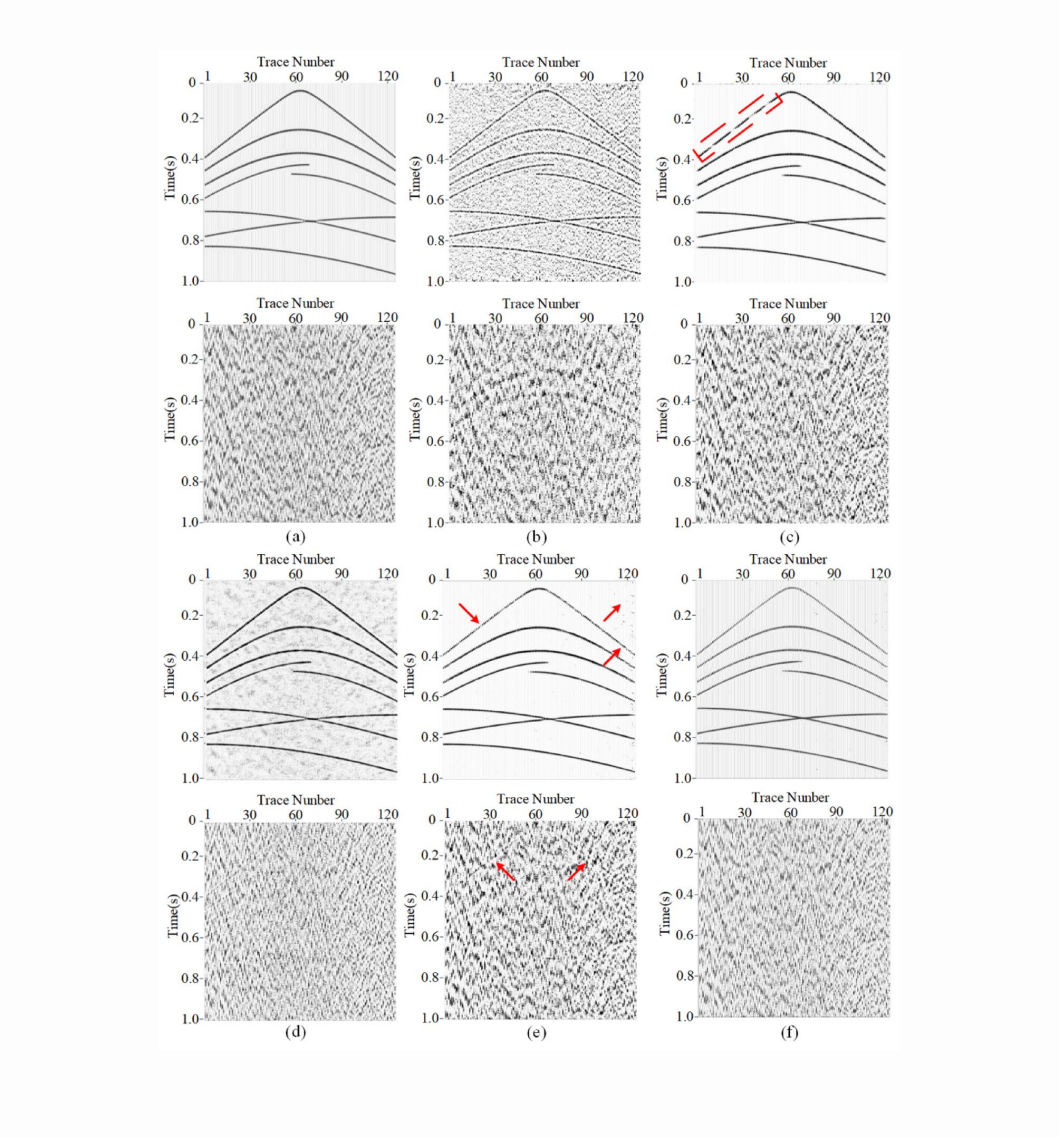
Comparison between denoising results obtained by different methods. **(a)** Clean record and added field noise. **(b)** to **(f)** Results (top subfigures) and removed noise (bottom subfigures) obtained by EEMD, Noiser2Noiser, B2U framework, MSAACNN and MSDPA-Net.

For further evaluating the denoisng performance, we have select some recently proposed denoising methods, including Noiser2Noiser^52^, EEMD^53^, MSAACNN^54^ and Blind2Unblind (B2U) denoising network (self-suprivised framework)^55^. Generally, EEMD is common used denoising methods in seismic data processing. Here, we set the ensemble number for the EEMD is 10. Moreover, MSAACNN are trained with the same training dataset and hyperparameter setting with those for MSDPA-Net. Meanwhile, we use the self-supervised training strategy to B2U network. Specifically, we use a mask to blind some information, and then use the coherence of reflection events to reconstruct the desired signals. Meanwhile, we also use Noiser2Noiser to adaptively attenuate the intense noise in un-supervised manner. The corresponding results are shown in Fig. 14.

By observing the figures, we can find that EEMD (Fig. 14(b)) fails to attenuate the intense aliasing seismic noise. Moreover, B2U framework shows limited performance in noise suppression. Generally, self-suprivised frameworks (Fig. 14(d)) usually utilize the differences between signal and noise components in temporal and spatial coherence to accomplish the denoising task. In specific, it is assumed that the signal is correlated and the noise is weakly correlated or uncorrelated^54^. However, the intense seismic background noise also represents potential correlation, which is inconsistent with the priori assumption. Furthermore, Noiser2Noiser (Fig. 14(c)) shows limited effects in the continuity and smoothness of recovered events, owing to the lack of labelled data^56,57^. Therefore, we can infer that these methods may not always provide reliable denoising performance. Meanwhile, by observing the contents marked by the red arrows, residual noise is existed in the denoising results of MSAACNN (Fig. 14(e)), and the reconstructed events are discontinuous in some areas. Compared with MSAACNN, MSDPA-Net (Fig. 14(f)) can more effectively attenuate the intense noise and recover the reflection signals.

## Conclusion

In this study, we designed a MSDPA-Net to address the low SNR and spectral aliasing in desert seismic data. Unlike traditional single-scale convolutional neural networks, MSDPA-Net employs a multi-scale strategy for preliminary feature extraction and introduces upsampling and downsampling units to effectively extract and utilize original feature information. Subsequently, in the dual-path attention module, the features are strengthened and differentiated by focusing on the original and different scales of the feature maps, respectively. Then, a feature interaction structure is used to reinforce the learning of signal and noise features. Finally, the feature reconstruction module is utilized to integrate and reorganize signal features to obtain the final denoising result. On the other hand, to ensure the denoising capability of MSDPA-Net, a training dataset is constructed using synthetic clean seismic records and real noise. The excellent performance of the trained model is verified by processing synthetic and field seismic records, demonstrating its significant advantages in recovering reflection signals in terms of amplitude and continuity even in strong interference environments, highlighting the unique ability of MSDPA-Net to preserve weak signals. Furthermore, the application of MSDPA-Net to field seismic records from different receiver lines further demonstrates its outstanding performance and versatility. While MSDPA-Net has yielded some promising results, it still requires enhancements in two key areas: denoising accuracy and generalization capability. These improvements are particularly necessary for handling seismic records with extremely low SNR. Nonetheless, MSDPA-Net carries significant value. It represents an interesting exploration into applying learning-based methods for attenuating intense seismic noise. Moreover, it could serve as an useful reference point for the development of other CNN frameworks.

## Supplementary Information

Below is the link to the electronic supplementary material.

Supplementary Material 1

## Data Availability

The datasets generated and analysed during the current study are not publicly available due to legal restrictions but are available from the corresponding author on reasonable request.
